# Draft Genome Sequence of *Mycobacterium asiaticum* Strain DSM 44297

**DOI:** 10.1128/genomeA.00320-14

**Published:** 2014-04-17

**Authors:** Olivier Croce, Catherine Robert, Didier Raoult, Michel Drancourt

**Affiliations:** Aix Marseille Université, URMITE, Marseille, France

## Abstract

We report the draft genome sequence of *Mycobacterium asiaticum* strain DSM 44297, a tropical mycobacterium seldom responsible for human infection. The genome of *M. asiaticum* has a size of 5,935,986 bp, with a 66.03% G+C content, encoding 5,591 proteins and 81 RNAs.

## GENOME ANNOUNCEMENT

*Mycobacterium asiaticum* is a nontuberculous mycobacterium initially described in 1971 (1) and recognized as an organism of medical interest in 1982 (2). *M. asiaticum* has been isolated in monkeys (3) and pigs (4). In humans, *M. asiaticum* isolates have been obtained from respiratory tract specimens (5, 6), lyphadenitis, bursitis, and wound specimens (7), and keratitis samples (8). In respiratory tract specimens, it is mainly recognized as a harmless contaminant but a few patients have been documented with *M. asiaticum* pneumonia (2, 9). Interestingly, this mycobacterium has been mainly documented in subtropical and tropical countries and areas, including Australia (7), California (9), Florida (8), Brazil (5), and Uganda (4).

In order to gain further insights into this organism, we performed whole-genome sequencing of the *M. asiaticum* DSM 44297 strain. Genomic DNA was extracted from mycobacteria grown on Middlebrook 7H10 agar medium at 37°C under a 5% CO_2_ atmosphere. A shotgun XL+ library and 3-kb paired-end library were pyrosequenced on a 454_Roche_Titanium platform (Roche-454 Life Sciences, Boulogne-Billancourt, France) (10). This project was loaded on a 1/4 region for each application on picotiter plates. The 454 sequencing generated 362,293 reads assembled into contigs and scaffolds using Newbler version 2.8 (Roche, 454 Life Sciences). Contigs obtained were combined together using Opera software v1.2 (11) and GapFiller v1.10 (12) to reduce the set. Finally, some manual refinements using CLC Genomics v5 software (CLC bio, Aarhus, Denmark) improved the genome. The draft genome sequence of *M. asiaticum* consists of 10 scaffolds of 106 contigs containing 5,910,460 bp, with an estimated genome size including gaps of 5,935,986 bp. Its G+C content is 66.03%.

Noncoding genes and miscellaneous features were predicted using RNAmmer (13), ARAGORN (14), Rfam (15), and PFAM (16). Open reading frames were predicted using Prodigal (17), and functional annotation was achieved using BLASTp against the GenBank database (18) and the Clusters of Orthologous Groups (COG) database (19, 20). The genome was shown to encode at least 81 predicted RNAs, including 3 rRNAs in a single operon, 51 tRNAs, 1 transfer-messenger RNA, and 26 miscellaneous RNAs. A total of 5,591 genes were also identified, representing a coding capacity of 5,458,539 bp (coding percentage: 91.9%). Among these genes, 742 (13.27%) were found to encode putative proteins and 881 (15.75%) were assigned as encoding hypothetical proteins. Moreover, 5,537 genes matched a least one sequence in the COG database with BLASTp default parameters.

### Nucleotide sequence accession numbers.

The *M. asiaticum* DSM 44297 strain genome sequence has been deposited at DDBJ/EMBL/GenBank under the accession no. HG964936 to HG964945. The whole-genome shotgun master numbers are CCBD010000001 to CCBD010000103.
